# Early Rod Dysfunction Influences Cone Development in a Rhodopsin P23H Mouse Model of Retinitis Pigmentosa

**DOI:** 10.3390/pathophysiology33010007

**Published:** 2026-01-14

**Authors:** Alicia A. Brunet, Annie L. Miller, Xin Ru Lim, Alan R. Harvey, Livia S. Carvalho

**Affiliations:** 1 Lions Eye Institute, 2 Verdun St., Nedlands, WA 6009, Australia; 2 Centre for Ophthalmology and Visual Sciences, The University of Western Australia, 35 Stirling Hwy, Crawley, WA 6009, Australia; 3 Department of Optometry and Vision Sciences, University of Melbourne, Parkville, VIC 3052, Australia; 4 School of Human Sciences, The University of Western Australia, 35 Stirling Hwy, Crawley, WA 6009, Australia; 5 Perron Institute for Neurological and Translational Science, 8 Verdun St., Nedlands, WA 6009, Australia

**Keywords:** retinitis pigmentosa, photoreceptor degeneration, P23H mouse, cone photoreceptor, retinal development, retinal compensation, inherited retinal disease

## Abstract

**Background/Objectives**: The *Rho^P23H/WT^* mouse line is a commonly used model to study rhodopsin P23H-associated autosomal dominant retinitis pigmentosa. Previous studies in *Rho^P23H/WT^* mice have largely focused on retinal changes occurring at one month of age and later, and have indicated a compensatory thickening of inner retinal layers in response to rod degeneration. However, the effect of disease processes during early postnatal retinal development remains understudied. **Methods**: In this study, we investigated the retinal response to rod dysfunction during early postnatal developmental ages P8–P24 in our novel *Rho^P23H/WT^* reporter line, *Rho*P23H.GFP, which expresses green fluorescent protein (GFP) exclusively in cone photoreceptors. **Results**: Histological analysis revealed no significant difference in retinal thickness in *Rho*P23H.GFP mice compared to healthy controls at the ages investigated. *Rho*P23H.GFP retinas initially exhibited a greater mislocalization of rhodopsin to the rod cell bodies at P12, though this mislocalization normalized to wildtype by P24. Most notably, flow cytometry revealed significantly increased cone photoreceptor numbers in P12 (61%), P16 (48%), and P24 (40%) *Rho*P23H.GFP mice compared to wildtype controls, indicating a possible compensatory response of cone photoreceptors to rod dysfunction. Additionally, cone morphology appeared altered in diseased cones. **Conclusions**: Our results suggest that cones may undergo a developmental compensatory adaptation in response to rod dysfunction, providing new insights into early disease mechanisms of retinitis pigmentosa.

## 1. Introduction

Retinitis pigmentosa is the leading cause of hereditary blindness, affecting around 1 in 4000 people worldwide [1], and it has over 80 disease susceptibility genes [2]. Retinitis pigmentosa is a progressive disease in which patients initially exhibit symptoms of night blindness followed by progressive loss of daylight vision [1]. Night blindness is usually a result of mutations causing the malfunction and degeneration of rod photoreceptor cells, which are required for low-light environments. Following night blindness is the progressive deterioration of vision from the periphery, extending centrally until central vision also begins to deteriorate, which may result in total blindness. Central vision loss is attributed to the secondary degeneration of the non-genetically compromised cones as a consequence of major rod degeneration [3].

One of the most common causes of autosomal dominant retinitis pigmentosa is the proline to histidine point mutation at codon 23 (P23H) in the rhodopsin gene (*RHO*) [4,5]. This single amino acid change accounts for approximately 10% of autosomal dominant retinitis pigmentosa cases [6]. Rhodopsin is the light-sensitive protein that initiates phototransduction in rod photoreceptors [7]. The P23H mutation has been extensively studied in animal models that mimic the disease, and it was shown to cause misfolding and aggregation of the rhodopsin protein in rod photoreceptors, leading to their degeneration [8,9,10,11]. Sakami and colleagues (2011) generated the *Rho* P23H mouse line, which is now a well-established and extensively used model to study the pathophysiological mechanisms of rhodopsin P23H-associated retinitis pigmentosa [12]. The heterozygous *Rho^P23H/WT^* mouse exhibits shorter rod outer segments by postnatal day 35 (P35) and half the number of rods by 2 months of age [12]. Interestingly, there is no apparent cone degeneration up to 5 months of age [12]. Recent work by Wang et al. (2024) revealed morphological preservation and compensatory retinal thickening of the inner nuclear layer of *Rho^P23H/WT^* mice at P28 and beyond [13]. However, whether such compensatory changes are occurring during early retinal development is unclear.

The aim of this study was to investigate the early retinal response to rod photoreceptor degeneration in *Rho^P23H/WT^* mice from P8 to P24, a critical window of retinal maturation. Retinal thicknesses of the outer, inner, and whole retina were measured. These were compared to healthy wildtype controls, as well as the retinal degeneration 1 (*rd1*) mouse, a model of autosomal recessive retinitis pigmentosa. Furthermore, we also investigated cone photoreceptor numbers between P12 and 6 months of age, providing new insight into cone photoreceptor developmental dynamics within retinitis pigmentosa.

## 2. Materials and Methods

### 2.1. Animals

This study was conducted in accordance with the ARVO Statement for the Use of Animals in Ophthalmic and Vision Research and was approved by the Harry Perkins animal ethics committee and the University of Western Australia ethics committee (AE198). The mouse lines used in this study were housed at the Harry Perkins Institute of Medical Research under a 12/12 h day/night cycle with ad libitum access to food and water. Mice used for this study ranged in age from P8 to 6 months old and were all on a C57BL/6J background. Mice were sacrificed via decapitation at P8 and P12, whilst P16 and above mice were euthanized via cervical dislocation. The Chrnb4.EGFP reporter line (STOCK Tg(Chrnb4-EGFP)CL200Gsat/Mmnc, RRID:MMRRC_000259-UNC) was obtained from the Mutant Mouse Resource & Research Centers (MMRRC) at the University of North Carolina at Chapel Hill, an NIH-funded strain repository, and was donated to the MMRRC by Nathaniel Heintz, Ph.D., The Rockefeller University, GENSAT. The Chrnb4.EGFP mouse expresses green fluorescent protein (GFP) in cone photoreceptors [14] and was used as the wildtype control in this study, and it is referred to as such from herein. The P23H rhodopsin knock-in mouse (The Jackson Laboratory, stock number 017628) [12] is an autosomal dominant model of retinitis pigmentosa and was crossbred with wildtype to generate heterozygous *Rho^P23H/WT^* mice. The retinal degeneration 1 (*rd1*) mouse model, which is a severe retinal degeneration model of autosomal recessive retinitis pigmentosa, was used as a comparison to the *Rho^P23H/WT^* mouse. The *rd1* mutations were isolated from the Chrnb4.GFP line as part of a previous C3H/HeJ background. The mutations were confirmed via genotyping that it carries the standard *Pde6b* mutations found in C3H/HeJ: the murine leukemia virus (Xmv-28) insertion in reverse orientation in intron 1 and a nonsense mutation (C-to-A transversion) in codon 347. Both retinitis pigmentosa models were crossbred with the Chrnb4.GFP wildtype mouse to generate GFP^+^ cones in all mouse lines (referred to as *Rho*P23H.GFP and *rd1*.GFP herein).

### 2.2. Immunohistochemistry

Frozen retinal sections for immunohistochemical staining were processed as follows. Eyes were marked for orientation, enucleated, and fixed in 4% *v*/*v* paraformaldehyde (PFA, #15710, Electron Microscopy Sciences, Hatfield, PA, USA) at room temperature for 1 h, with the cornea and iris being removed after 30 min fixation. After fixing, eyes were then cryoprotected overnight in 20% *w*/*v* sucrose solution at 4 °C. The lens was removed, and the remaining eyecup was embedded in an optimal cutting temperature (#4583, Tissue-Tek, Torrance, CA, USA) compound, frozen with isopentane (#320404, Sigma-Aldrich, Burlington, MA, USA), and submerged in liquid nitrogen. Eyecups were sectioned on a cryostat (CM3050S, Leica, Wetzlar, Germany) at 14 µm, and sections were placed on frosted microscope slides (SCF90W-PC, Hurst Scientific, Forrestdale, WA, Australia). Sections were blocked for 1 h in blocking buffer (1% *w*/*v* bovine serum albumin [BSA; BSAS0.1, Bovogen Biologicals, Keilor East, VIC, Australia], 0.5% *v*/*v* Trion-X-100 [1552–500 mL, LabChem, Zelienople, PA, USA], 5% *v*/*v* normal goat serum [NGS; G9023, Sigma-Aldrich, Burlington, MA, USA], in 1× PBS) for 1 h at room temperature. Primary antibodies diluted in blocking buffer were added to the sections and were incubated overnight at 4 °C. Primary antibodies used in this study include rhodopsin (1:1000, ab3424, Abcam, Cambridge, UK), which stains for rhodpsin protein within rod photoreceptors [15], and peanut agglutinin (PNA; conjugated to Alexa Fluor 568 1:500, L32458, Life Technologies, Carlsbad, CA, USA), which stains cone photoreceptor sheaths to visualize cone inner and outer segments [16]. Following overnight incubation, sections were washed three times with 1× PBS before the addition of a secondary antibody for rhodopsin staining (Alexa Fluor 568 1:500, ab175471, Abcam, Cambridge, UK) diluted in blocking buffer (2 h at room temperature). After incubation with the secondary antibody, sections were washed three times with 1× PBS and incubated in sudan black (#199664, Sigma-Aldrich, Burlington, MA, USA) for 30 min at room temperature to limit tissue autofluorescence. Finally, sections were counterstained with 4′,6-diamidino-2-phenylindole (DAPI) for 5 min at room temperature and imaged on a Nikon AX confocal microscope (Nikon, Shinagawa, Japan).

### 2.3. Histological Analysis

To eliminate bias, histological slides with frozen sections for retinal thickness quantification were randomly blinded by one investigator prior to imaging by another investigator. A Nikon Eclipse Ni fluorescent microscope (Nikon, Shinagawa, Japan) with a 20× dry objective was used to capture quantitative images for retinal thickness, with each biological replicate having two adjacent retinal sections imaged. Each section had four positions imaged relative to the optic nerve: +80° (superior peripheral, SP), +10° (superior central, SC), −10° (inferior central, IC), and −80° (inferior peripheral, IP). The thickness of the outer nuclear layer (ONL), inner nuclear layer (INL), and whole retinal thickness (top of the ONL to the ganglion cell layer) was manually measured from each region by averaging four equidistant measurements within each image using the NIS-elements AR 5.42.01 software (Nikon, Shinagawa, Japan). Individual images were not quantified if the retinal section was too damaged to provide an accurate measurement. Outlier tests were performed and found one outlier for retinal thickness measurements of a WT mouse at P8, which was removed from the final results, resulting in *n* = 4. Representative images were taken using a Nikon AX confocal microscope (Nikon, Shinagawa, Japan) on a 20× dry objective for increased image resolution compared to initial quantification images.

### 2.4. Cone Photoreceptor Quantification

Fresh retinas were dissected and dissociated with a papain digestion. A papain/DNase solution (1:20, LK003170/LK003176, Worthington Biochemicals, Lakewood, NJ, USA) was made to 100 µL/sample and activated at 37 °C on a heat block for 30 min before adding the dissected retina. Samples were then incubated for 30 min at 37 °C and gently triturated with a pipette to completely dissociate the tissue. A total of 200 µL of inhibitor solution (Earle’s balanced salt solution [EBSS; #24010-043, Gibco, Waltham, MA, USA], 9.5% *v*/*v* ovomucoid protease inhibitor [OMI; LK003182, Worthington Biochemicals, Lakewood, NJ, USA], 5% *v*/*v* DNaseI [LK003170, Worthington Biochemicals, Lakewood, NJ, USA]) was added to each sample and was incubated at 37 °C for 10 min. The sample was then spun at 1400× rpm for 5 min before being resuspended again in 300 µL resuspension solution (EBSS, 10% *v*/*v* DNaseI). eBioscience™ Fixable Viability Dye eFluor™ 660 (#65-0864-14, Invitrogen, Waltham, MA, USA) was used as a dead cell stain and was added at a 1:1000 dilution after resuspension of the samples and incubated on ice in the dark for 30 min. Fluorescent activated cell sort (FACS) buffer (2% heat-inactivated fetal calf serum [HI-FCS; WS-FBS-AU0-015, Fisher Biotec, Perth, WA, Australia], 1 mM ethylenediaminetetraacetic acid [EDTA], 1× PBS) was added at 1 mL/sample to wash off the cell stain after incubation, and the sample was spun down at 1400× rpm for 5 min to collect the pellet. The pellet was resuspended in 500 µL FACS buffer. Once retinal samples were dissociated, total cell numbers were analyzed on a Z Series Coulter Counter (Beckman Coulter, Brea, CA, USA). Flow cytometry was performed on the BD FACSMelody™ Cell Sorter (BD Bioscience, Franklin Lakes, NJ, USA) to quantify the percentage of GFP^+^ cells in each sample, representing the percentage of cone photoreceptors.

### 2.5. Statistical Analysis

Statistical analysis was conducted on GraphPad Prism software v9.5.1. Data was tested for Gaussian distribution using a Shapiro–Wilk test. Data followed normal distribution, and two-way ANOVA and Sidak’s multiple comparisons test were used, with statistical significance being identified as *p* < 0.05. All data are presented as mean ± standard error of the mean (SEM).

## 3. Results

**Figure 1:**
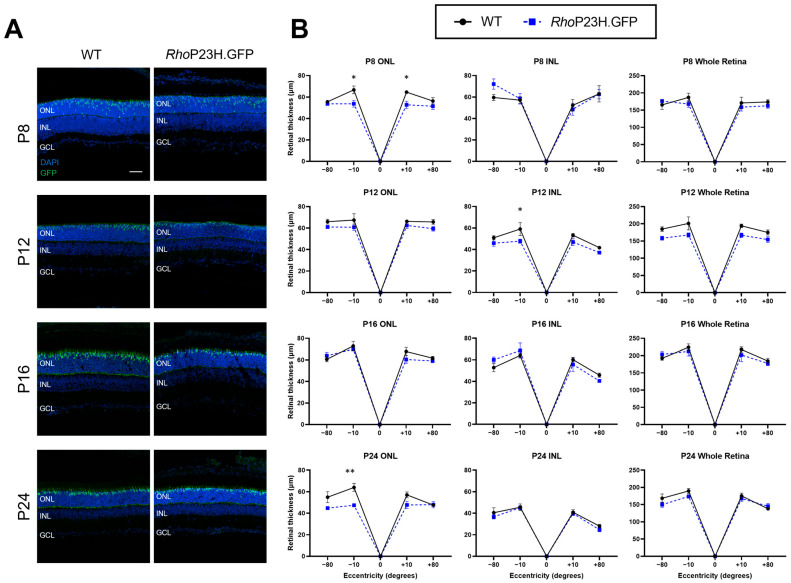
Retinal thickness of *Rho*P23H.GFP mice compared to wildtype controls at P8, P12, P16, and P24. (**A**) Representative retinal thickness histology images. Images were taken of the medial retina and show cell nuclei with a DAPI stain (blue) and cone photoreceptors (green). ONL: outer nuclear layer; INL: inner nuclear layer; GCL: ganglion cell layer. Scale bar = 50 µm. (**B**) Retinal thickness quantification of the ONL, INL, and whole retina. Numbers are mean ± SEM. Each *n* = one retina per animal. WT: P8 *n* = 4, P12 *n* = 4, P16 *n* = 4, P24 *n* = 4; *Rho*P23H.GFP: P8 *n* = 4, P12 = *n* = 4, P16 *n* = 4, P24 *n* = 5. * *p* < 0.05, ** *p* < 0.01.

Retinal degeneration due to the heterozygous rhodopsin P23H mutation is considered to occur relatively slowly, as only one rhodopsin allele is dysfunctional whilst the other allele is still generating functional wildtype rhodopsin protein [9,12]. As such, studies in P23H mice have had a focus on characterizing the model at 1 month of age and above [11,12,13]. Figure 1 shows representative histology images and quantification of retinal thickness in the outer nuclear layer (ONL), inner nuclear layer (INL), and whole retina of *Rho^P23H/WT^* mice compared to wildtype during early ages P8, P12, P16, and P24. The mouse lines generated for this study all express green fluorescent protein (GFP) in cone photoreceptors for ease of their differentiation from rod photoreceptors and are referred to as .GFP at the end of their mouse line name (for example, *Rho*P23H.GFP). There were no major differences in the retinal thickness consistently across all retinal regions measured between *Rho*P23H.GFP mice compared to wildtype controls, indicating minimal retinal degeneration occurring in *Rho*P23H.GFP mice from P8 to P24. However, there was a significant decrease in ONL thickness in central regions at P8 but not at P12 and P16 in *Rho*P23H.GFP, which is then significantly decreased again at P24 in the inferior central retina. There was also a significant decrease at P12 in the inferior central INL.

**Figure 2:**
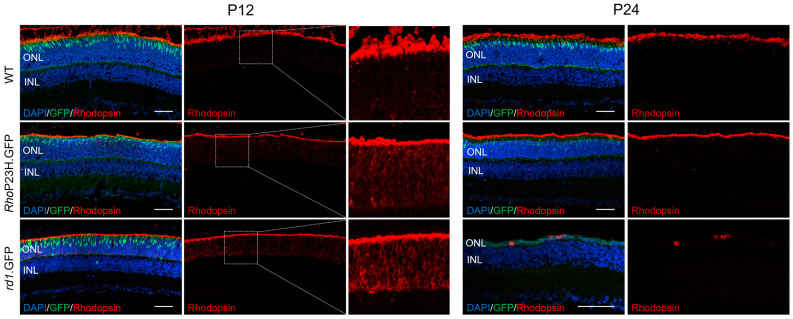
Rhodopsin protein expression at P12 and P24 in wildtype, *Rho*P23H.GFP, and *rd1*.GFP mice. Representative immunohistochemistry images are shown of the medial retina in all mouse lines of cell nuclei (blue), rhodopsin (red) and cones (green). Insets are zoomed-in images of the ONL with increased fluorescence intensity for identification of mislocalized rhodopsin. ONL: outer nuclear layer; INL: inner nuclear layer; GCL: ganglion cell layer. Scale bar = 50 µm.

Previous studies have indicated that mutant rhodopsin is mislocalized to the cell body and inner segments in some P23H models [10,17,18], though interestingly, in the Sakami et al. (2011) variation of this model, rhodopsin mislocalization is not reported [11]. We characterized rhodopsin expression during early retinal development (P12) in Figure 2. Rhodopsin expression was also compared to another retinitis pigmentosa model, the autosomal recessive *rd1* mouse that carries mutations in the *Pde6b* gene [19], to assess whether aberrant expression of rhodopsin was specific to the P23H mutation or whether it was a general effect of mutations in rod-specific phototransduction genes. Rhodopsin was localized to both the outer segment and ONL in all models at P12, though this was more apparent in the disease models compared to wildtype as a possible result of rod-specific mutations. By P24, rhodopsin protein was correctly localized to the outer segment in *Rho*P23H.GFP mice. Due to the severe photoreceptor degeneration and outer segment loss in *rd1*.GFP mice, rhodopsin staining at P24 was localized to the remaining ONL cell bodies.

**Figure 3:**
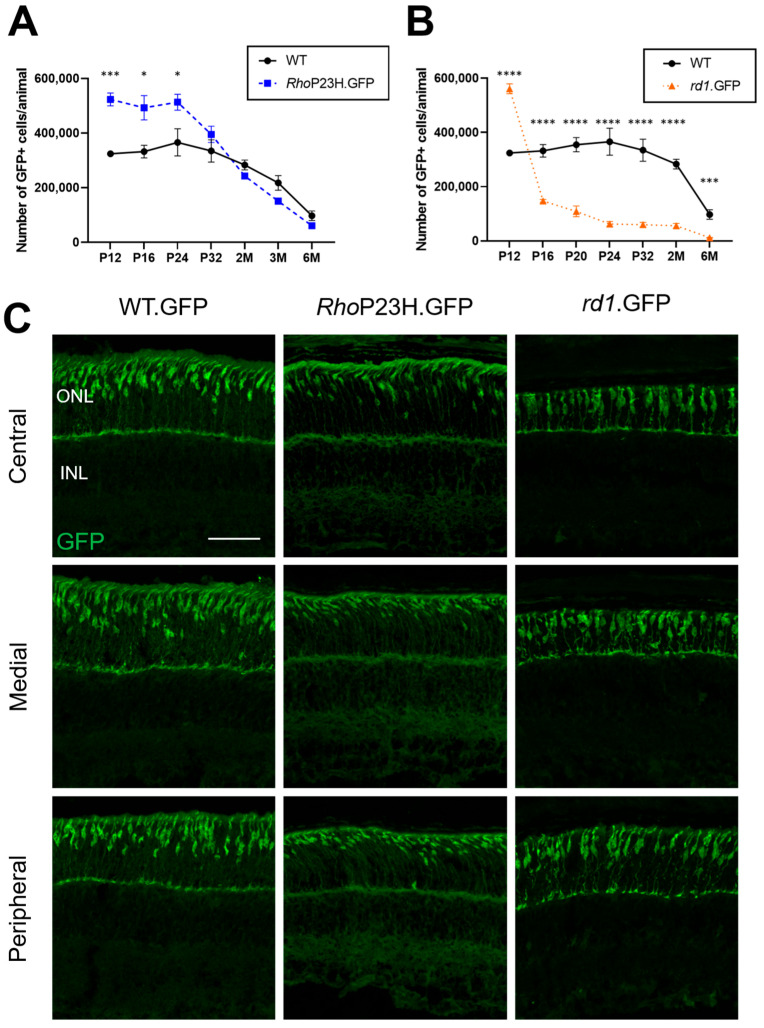
Cone photoreceptor numbers and morphology in retinitis pigmentosa mice and wildtype. Cone numbers were quantified for (**A**) *Rho*P23H.GFP and (**B**) *rd1*.GFP mice compared to age-matched wildtype controls. Numbers are mean ± SEM. Each *n* = two retinas per animal. WT: P12 *n* = 4, P16 *n* = 4, P20 *n* = 4, P24 *n* = 8, P32 *n* = 6, 2M *n* = 4, 3M *n* = 4, 6M *n* = 6; *Rho*P23H.GFP: P12 *n* = 6, P16 *n* = 3, P24 *n* = 4, P32 *n* = 4, 2M *n* = 3, 3M *n* = 4, 6M *n* = 4; *rd1*.GFP: P12 *n* = 3, P16 *n* = 5, P20 *n* = 4, P24 *n* = 8, P32 *n* = 4, 2M *n* = 3, 6M *n* = 3. * *p* < 0.05, *** *p* < 0.001, **** *p* < 0.0001. 2M: 2 months; 3M: 3 months; 6M: 6 months. (**C**) Representative immunohistochemistry images of cone photoreceptors (green) in the central, medial, and peripheral retina at P12. ONL = outer nuclear layer; INL = inner nuclear layer. Scale bar = 50 µm.

As we did not see widespread rod degeneration in the *Rho*P23H.GFP mouse compared to wildtype controls, we also assessed whether cone photoreceptors were affected at these early ages. Despite the presence of rod degeneration in adult *Rho^P23H/WT^* mice, previous studies have indicated that cone numbers are seemingly unaffected when quantified from 1 month of age onwards [12,20]. One could then assume that cone numbers were similar to wildtype at ages earlier than 1 month, though this has not been previously explored. As cones in our lines are GFP-labeled, we performed flow cytometry to estimate cone numbers (Figure 3A,B). Strikingly, at early postnatal timepoints, *Rho*P23H.GFP had a significantly higher number of cones compared to wildtype up to P24. Cone numbers in *Rho*P23H.GFP mice were elevated relative to wildtype controls by 61% at P12, 48% at P16, and 40% at P24 (Supplementary Table S1). We also assessed cone numbers in *rd1*.GFP mice, and they similarly displayed a significantly higher number of cones at P12 compared to wildtype mice (73% increase), which drastically dropped by P16 (55% decrease). Representative images of cone photoreceptors at P12 in different retinal regions are also shown in Figure 3C. The cone morphology in *Rho*P23H.GFP mice appear to have a slanted appearance compared to wildtype, which was identified in all samples we investigated at P12.

**Figure 4:**
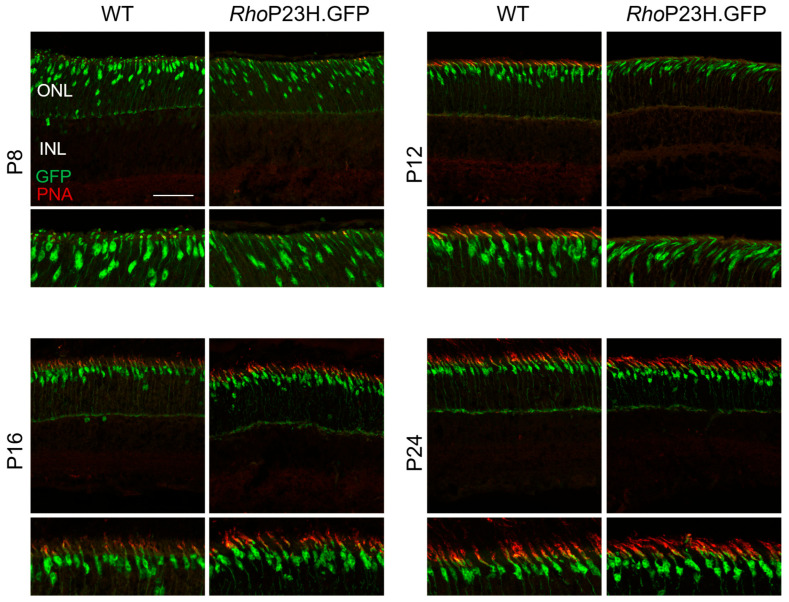
Cone outer segment morphology in wildtype and *Rho*P23H.GFP mice across different ages. Representative immunohistochemistry images of cone photoreceptors (green) with PNA staining (red) to show outer segments. Images were taken from the medial retina at P8, P12, P16, and P24. ONL = outer nuclear layer; INL = inner nuclear layer. Scale bar = 50 µm.

Given potential cone morphology changes, we assessed whether cone outer segments were affected at early stages of retinal development using peanut agglutinin (PNA) staining (Figure 4). At P8, cone outer segments are beginning to form, with PNA only being visible in wildtype from P12 onwards. Cone outer segments were potentially more slanted in appearance in *Rho*P23H.GFP mice compared to wildtype from P12 and showed delayed expression of PNA from P16 onwards only.

## 4. Discussion

This study investigates early postnatal retinal changes in our novel reporter model of the *Rho^P23H/WT^* mouse (*Rho*P23H.GFP) from P8 to P24. While previous studies in *Rho^P23H/WT^* mice have primarily focused on retinal degeneration at later stages (around 1 month and beyond), we report on the early-stage retinal features not previously characterized within this disease model. In contrast to previous studies, we did not find a compensatory response of thickening of the inner retinal layers to photoreceptor degeneration. However, our findings did reveal a compensatory adaptation of cone photoreceptors, in which cone numbers were significantly increased compared to wildtype controls, as well as potential delayed cone outer segment formation. These findings suggest a compensatory adaptation of cone photoreceptors before the onset of major rod degeneration and provide new insight into the cellular changes occurring in early retinitis pigmentosa progression.

### 4.1. Characterization of Retinal Thickness and Rhodopsin Localization in RhoP23H.GFP Mice During Early Retinal Development

The findings by Wang et al. (2024) of increased inner retinal thickness in *Rho^P23H/WT^* at P28 and beyond [13] prompted our investigation of early degeneration events up to P24. We did not observe significant thinning of the outer or inner nuclear layers via histology. In contrast to previous findings, we found retinal thickness to be mostly similar between disease and wildtype mice at P8, P12, P16, and P24, indicating that widespread rod loss had not yet occurred at these ages. One limitation of investigating retinal integrity using histology is that the retinal tissue is subject to histological processing artifacts compared to optical coherence tomography (OCT), which assesses retinal thickness in vivo. This may explain the disparity in the findings between our study and Wang et al. (2024), which was due to methodological differences, since they measured INL via OCT [13]. A study by Monai et al. (2018) found that OCT was more sensitive to early photoreceptor inner segment and outer segment ultrastructural changes in a P23H rat model [21], though whether these two methods rendered different retinal thickness measurements was not evaluated. Another possible confounding factor could be our use of a transgenic GFP P23H mouse model. However, whether this would significantly affect retinal thickness measurements is unlikely due to the expression of GFP being restricted to cone photoreceptors. Furthermore, we have previously validated that the transgenic IRD cone GFP models generated within our lab do not incur major retinal changes as a result of the transgene expression [22].

Unlike some rodent variants of the P23H model in which misfolded rhodopsin accumulates in the inner segments and cell bodies, our data showed that rhodopsin was largely localized correctly to the rod outer segments by P24 in *Rho*P23H.GFP mice. This aligns with the original Sakami et al. (2011) line generation paper [12]. Interestingly, there was a greater mislocalization of rhodopsin at the crucial timepoint of eye opening (P12) in *Rho*P23H.GFP mice compared to wildtype, though this was also found in *rd1*.GFP mice. These findings suggest that rhodopsin mislocalization is not limited to mutations within the rhodopsin gene and may be from mutations in rod-specific genes in general. Interestingly, it seems that light and activation of the phototransduction cascade after eye opening may be positively impacting rhodopsin localization.

### 4.2. Cone Compensatory Adaptation in Response to Defective Rods

One of the most striking findings from this study is the significant increase in cone photoreceptor numbers in *Rho*P23H.GFP mice compared to wildtype during early retinal postnatal development. Our findings indicate a significant increase in cone numbers until P24, raising the possibility that the early postnatal retinal environment in *Rho*P23H.GFP retinas promote a compensatory response in cones. This may reflect an attempt by the developing retina to preserve visual capacity in the event of rod loss or dysfunction. By P32, cone numbers were similar to wildtype. This timeline matches previously identified rod changes where outer segment morphology is significantly altered by P35 [12], possibly suggesting that cone numbers in *Rho*P23H.GFP mice only begin to normalize once rod degeneration is evident. Whether these findings lead to functional consequences on cone-mediated vision is still unknown.

Interestingly, we also show that these compensatory changes are also present in the *rd1.*GFP mice, which undergo rapid and severe rod degeneration commencing around P8 [23], and have elevated cone numbers at P12 before sharply declining at P16. This temporal pattern suggests that early increases in cone numbers are not limited to one form of retinitis pigmentosa and may be a general response to rod photoreceptor stress, regardless of the inheritance pattern. The sustained response in elevated cone numbers in *Rho*P23H.GFP mice until P24, in contrast to the rapid decline in *rd1.*GFP mice after P12, suggests that the slower rod degeneration in *Rho*P23H.GFP may be providing a longer window for an adaptive cone response. However, this study only examined two mouse models of retinitis pigmentosa, and it remains unclear whether this compensatory increase in cone numbers occurs in other models of the disease. Furthermore, it would be valuable to explore whether similar early cone adaptations occur in other inherited rod–cone dystrophy models and whether these compensatory changes translate to the human disease presentation.

Several potential mechanisms could explain this initial increase in cone numbers. One possibility is a transient delay or reduction in the normal process of developmental pruning of cone photoreceptors. If early rod dysfunction alters this process, for instance, by disrupting cell death signaling or changes to neurotrophic support, it could result in preservation of cones that would otherwise be eliminated. Future studies could perform terminal deoxynucleotidyl transferase-dUTP nick end labeling (TUNEL) staining, which labels dying cells, to determine whether TUNEL^+^ cells colocalize with GFP^+^ cones to assess whether there is a lack of cone photoreceptor cell death in *Rho*P23H.GFP mice compared to healthy mice during early retinal development. Another theory could be that more cones are born during embryonic development as a result of intrinsic transcriptional changes in photoreceptor fate determination. Retinal progenitor cells are programmed to become cone photoreceptors by default unless rod-promoting factors, *Nrl* and *Nr2e3*, are expressed to suppress cone fate [24]. This could be evaluated using retinal cell lineage tracing during early embryonic development, in which cone-producing progenitors are labeled, using genes such as *Otx2* or *Onecut1* [25], to determine whether a greater proportion of these progenitor cells adopt a cone photoreceptor fate in the *Rho*P23H.GFP retina. Despite these hypotheses, it is currently unclear whether mutations in the rhodopsin gene or other retinitis pigmentosa-associated genes may affect embryonic cell fate determination.

In addition to increased cone numbers in *Rho*P23H.GFP, there may be a delay in cone outer segment formation as identified by decreased PNA expression until P16, and whether the two adaptations are linked could be postulated. Light is known to play an important role during retinal development, such as synapse formation, cone organization, and photoreceptor outer segment development [26,27,28]. The absence of light in dark-reared *rd10*, another mouse model of retinitis pigmentosa, has shown to protect photoreceptors from degeneration [29]. Perhaps this delay in outer segment formation in *Rho*P23H.GFP protects cones from degeneration during early retinal formation as a way of avoiding light activation. A previous study has shown that *Rho^P23H/WT^* cone-mediated electroretinogram (ERG) responses are hypersensitive to light around one and three months of age, and single-cell recordings of the primary visual cortex revealed spontaneous firing of V1 neurons in photopic conditions [30]. This hypersensitive photopic ERG response could also be present during early retinal development due to our finding of increased cone numbers and would be of interest to explore further.

The compensatory adaptation of cones is not a completely unexpected finding, as other retinal adaptations have been identified in P23H-affected mice. Leinonen et al. (2020) have suggested that aspects of rod-mediated vision are preserved through potentiation of the rod-to-rod bipolar signal transmission in *Rho^P23H/WT^* mice [20]. The same study conducted RNA-seq analysis on total extracted RNA and found upregulation of pathways associated with synaptic organization and axonogenesis. It would be of great interest for future research to assess the single-cell transcriptomic changes that occur in cone photoreceptors that facilitate these adaptation changes during early development. Retinal remodeling has been identified in autosomal recessive retinitis pigmentosa models, including *rd1* [31] and *rd10* [32] mice, as cones form ectopic synapses with rod bipolar cells. This remodeling, however, was only evident once rod degeneration had commenced [33].

## 5. Conclusions

In summary, this study reveals early changes in cone photoreceptors in *Rho*P23H.GFP mice during postnatal development, including increased cone numbers and altered morphology, in the absence of substantial overall retinal thinning within this model between P8 and P24. These findings challenge whether cones remain unaffected during early retinitis pigmentosa progression before major rod degeneration and may suggest cones undergo compensatory adaptation to maintain increased numbers at early ages. If this phenotype also occurs in the human presentation of *RHO*-associated retinitis pigmentosa, it may reveal a previously unrecognized therapeutic window during early disease progression that could be targeted to delay cone degeneration in patients. Identifying these compensatory responses provides new insights into understanding early retinitis pigmentosa disease progression.

## Data Availability

The data collected in the current study are available from the corresponding author upon request.

## Supplementary Materials

The following supporting information can be downloaded at https://www.mdpi.com/article/10.3390/pathophysiology33010007/s1, Table S1: Percentage change in cone numbers between retinitis pigmentosa mouse models and age-matched wildtype controls.

## Abbreviations

The following abbreviations are used in this manuscript:

GFP	Green fluorescent protein
*rd1*	Retinal degeneration 1
PFA	Paraformaldehyde
BSA	Bovine serum albumin
NGS	Normal goat serum
PBS	Phosphate-buffered saline
PNA	Peanut agglutinin
DAPI	4′,6-diamidino-2-phenylindole
SP	Superior peripheral
SC	Superior central
IC	Inferior central
IP	Inferior peripheral
ONL	Outer nuclear layer
INL	Inner nuclear layer
EBSS	Earle’s balanced salt solution
OMI	Ovomucoid inhibitor
FACS	Fluorescent-activated cell sort
HI-FCS	Heat-inactivated foetal calf serum
EDTA	Ethylenediaminetetraacetic acid
OCT	Optical coherence tomography
