# Supplementary material for: Early Rod Dysfunction Influences Cone Development in a Rhodopsin P23H Mouse Model of Retinitis Pigmentosa
Source: Pathophysiology. 2026 Jan 14;33(1):7. doi: 10.3390/pathophysiology33010007 (PMC12821531; doi:10.3390/pathophysiology33010007)
Supplement: Supplementary file 1 [file pathophysiology-33-00007-s001.zip › pathophysiology-3943681-supplementary.pdf]

Supplementary Table S1. Percentage change in cone numbers between retinitis pigmentosa mouse models and age-matched wildtype controls.

**Age**	**WT**	***Rho*P23H.GFP**		***rd1*.GFP**	
	Average cone numbers	Average cone numbers	Percentage change	Average cone numbers	Percentage change
P12	324370 ± 8389	523224 ± 23280	61.3 ↑	561487 ± 17996	73.1 ↑
P16	332347 ± 23055	492831 ± 44357	48.3 ↑	147377 ± 5249	55.7 ↓
P20	354875 ± 26151	*not measured*		108965 ± 19817	69.3 ↓
P24	365933 ± 49754	513033 ± 29121	40.2 ↑	62248 ± 9162	83.0 ↓
P32	334677 ± 40682	395351 ± 29841	18.1 ↑	59842 ± 8537	82.1 ↓
2M	283272 ± 17896	242571 ± 8491	14.4 ↓	55638 ± 8475	80.4 ↓
3M	217512 ± 26852	150749 ± 11400	30.7 ↓	*not measured*	
6M	97478 ± 17500	60570 ± 4927	37.9 ↓	11557 ± 1156	88.1 ↓

↑ indicates increased change ↓ indicates decerased change
